# Breast cancer gene expression datasets do not reflect the disease at the population level

**DOI:** 10.1038/s41523-020-00180-x

**Published:** 2020-08-25

**Authors:** Yanping Xie, Brittny C. Davis Lynn, Nicholas Moir, David A. Cameron, Jonine D. Figueroa, Andrew H. Sims

**Affiliations:** 1 Applied Bioinformatics of Cancer, University of Edinburgh Cancer Research Centre, MRC Institute of Genetics and Molecular Medicine, Edinburgh, UK; 2 Usher Institute, University of Edinburgh, Edinburgh, UK; 3 Division of Cancer Epidemiology and Genetics, National Cancer Institute, Bethesda, MD USA; 4 NHS Research Scotland Cancer Lead and Cancer Research UK Edinburgh Centre, MRC Institute of Genetics & Molecular Medicine, The University of Edinburgh, Edinburgh, UK

**Keywords:** Cancer genomics, Cancer epidemiology

## Abstract

Publicly available tumor gene expression datasets are widely reanalyzed, but it is unclear how representative they are of clinical populations. Estimations of molecular subtype classification and prognostic gene signatures were calculated for 16,130 patients from 70 breast cancer datasets. Collated patient demographics and clinical characteristics were sparse for many studies. Considerable variations were observed in dataset size, patient/tumor characteristics, and molecular composition. Results were compared with Surveillance, Epidemiology, and End Results Program (SEER) figures. The proportion of basal subtype tumors ranged from 4 to 59%. Date of diagnosis ranged from 1977 to 2013, originating from 20 countries across five continents although European ancestry dominated. Publicly available breast cancer gene expression datasets are a great resource, but caution is required as they tend to be enriched for high grade, ER-negative tumors from European-ancestry patients. These results emphasize the need to derive more representative and annotated molecular datasets from diverse populations.

## Introduction

An ever-increasing number of cancer transcriptomics datasets are now publicly available enabling researchers to perform highly informative retrospective gene expression analysis. The majority of these transcriptome microarray and RNAseq datasets are still relatively small, limited to 20–300 patients, except for large consortia studies such as TCGA^1^ and METABRIC^2^. Even relatively small studies can be repurposed to provide valuable insights, particularly if they have detailed information on the tumor and patient characteristics. Gene expression data from functional studies in cancer cell lines or in vivo experiments can be compared with clinical datasets to evaluate the reliability of model systems to recapitulate the disease. These clinical datasets can also be used to assess associations between putative oncogenes or tumor suppressors and different signaling pathways or clinical characteristics to examine whether certain subgroups of tumors have elevated or reduced expression of particular genes.

To better understand the variability and the extent to which these datasets represent breast cancers at the population level, a comprehensive analysis of molecular subtypes and prognostic signatures across all publicly available datasets was performed. A total of 70 breast cancer gene expression datasets representing 16,130 patients were examined. Limited patient and tumor information was provided for many of studies and wide variations in molecular composition were uncovered between the cohorts. Publicly available datasets were found to be biased towards high grade, ER-negative tumors from European-ancestry patients compared with the wider population.

## Results

### Limited annotation of published gene expression datasets

Availability of clinical annotation associated with each dataset was limited and highly variable, from reasonably complete information on country of origin (79%) and decade of diagnosis (68%), to more sparse details on characteristics such as age at diagnosis (30%), grade (49%), tumor size (37%) to very scant information on characteristics like BMI (5%). Molecular subtype assignments were similar using different classifiers. The PAM50 method was most concordant across platforms, assessed by 573 replicate tumors processed by RNAseq and Agilent microarray data from the TCGA study (Supplementary Fig. 1). The PAM50 and Mammaprint risk of relapse estimated scores were also highly consistent, although Mammaprint correlations were a little lower (R^2^ = 0.8) than for PAM50 scores (R^2^ = 0.9).

### Wide variation in molecular composition of studies

**Fig. 1:**
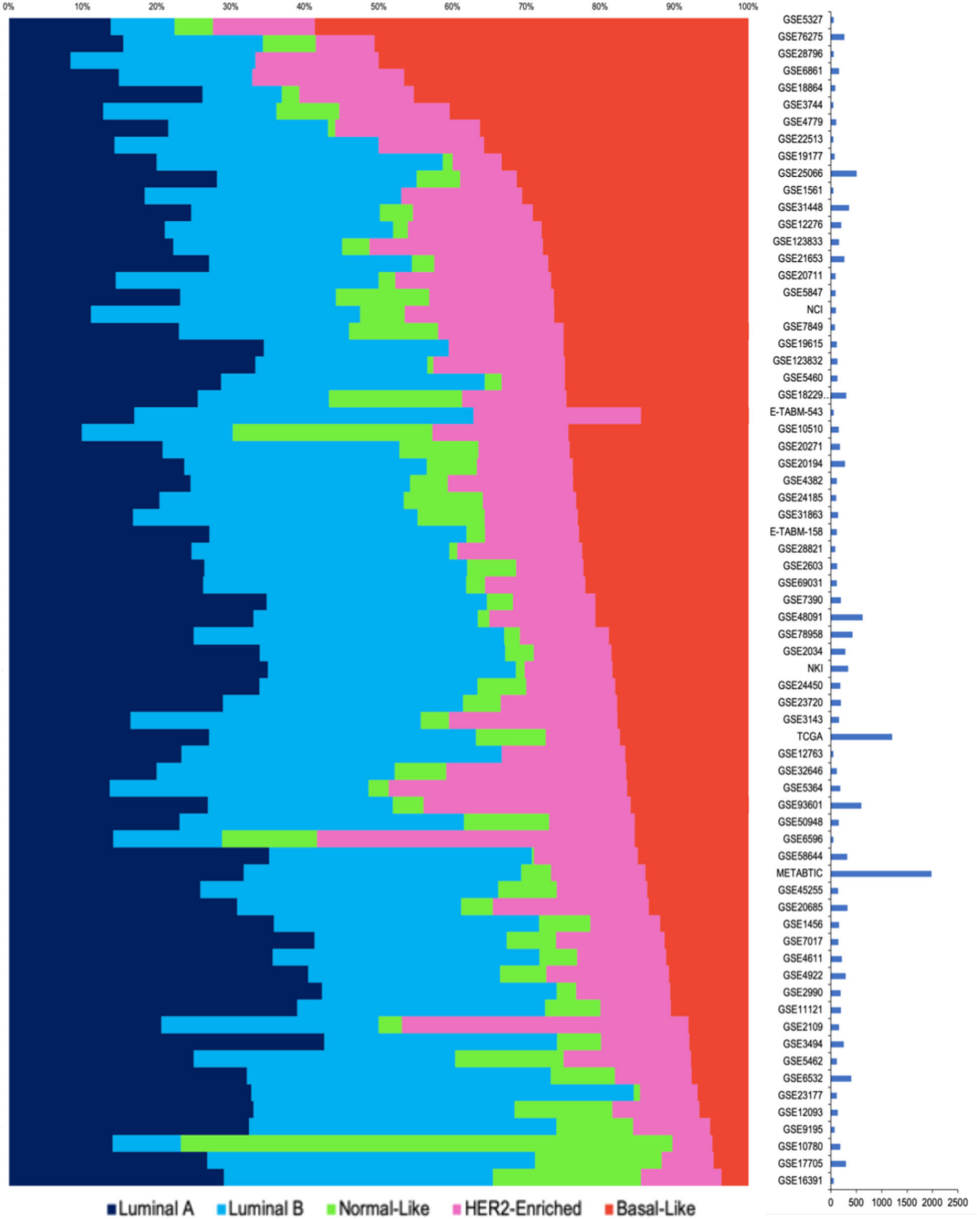
Molecular subtypes of breast cancer are highly variable across publicly available datasets. Distributions of PAM50 intrinsic subtypes assigned by the geneFu package across 70 datasets. The median proportion of luminal A tumors was 25% and luminal B was 31%. Some datasets were dominated by specific subtypes and some were completely lacking in normal-like breast tumors.

**Fig. 2:**
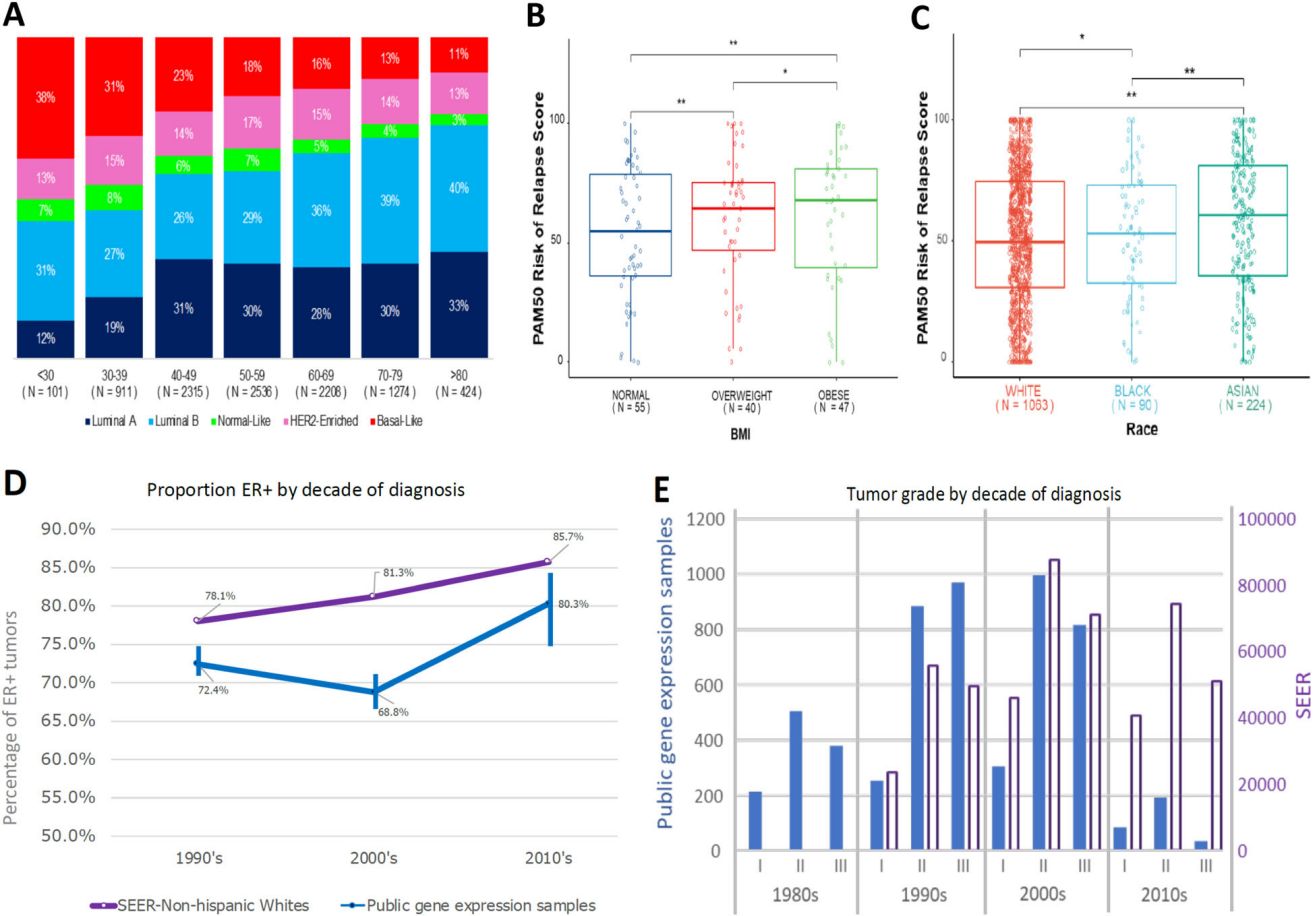
Publicly available breast cancer gene expression datasets recapitulate some epidemiological trends, but have reduced proportions of ER+ and grade 1 tumors compared with Western populations. The distribution of molecular subtypes by age (**a**) and the association between BMI and molecular predictions of poor outcomes (**b**) are as would be expected. However, Asians older than 50 appear to have worse predicted prognosis than other races (**c**), but this is likely confounded by other factors. The boxes represent upper and lower quartile ranges, horizontal line the median and whiskers indicate 1.5× the interquartile range. Incidence rates for ER-positive tumors progressively increase overtime, but the proportion remains significantly lower than that reported by SEER (**d**), vertical bars represent 95% confidence intervals. Grade 3 tumors were most abundant in publicly available datasets for the 1990s, which does not reflect SEER figures, which show increasing proportions of grade 1 tumors over the last three decades (**e**).

The relative proportions of molecular subtypes varied widely between datasets, with basal tumors ranging from 4 to 59% (Fig. 1). Overall the proportion of basal subtype or ER-negative (35%) tumors was considerably higher than the 10% HR-/HER2-observed across broader, unselected populations from the SEER database 2007–2013^3^. It is noteworthy that one dataset (GSE10780) was dominated by ‘normal-like’ tumors, which were absent from several others (e.g., GSE28796, GSE6861, GSE22513, and GSE19615). There appear two possible reasons for these observations, both a genuine preeminence and absence of minimally aberrant tumors, or simply that breast tumors in these datasets had particularly low/high tumor content, given that doubts remain over the existence of normal-like subtypes^4^. The proportion of basal-like tumors significantly decreases with age (Fig. 2a) as has been shown before in the large American Cancer Society study^5^.

### Some population level epidemiological associations are evident, but others appear distorted

PAM50 risk of relapse scores for those patients who were younger than 50 years old and overweight or obese patients were significantly higher than those of normal weight patients (Fig. 2b), consistent with expectations of the association between BMI and poor outcomes^6^. Not all trends were as expected, the estimated PAM50 prognostic scores of Asian patients over 50 years old were significantly higher than for Whites (*p* < 0.01) and Blacks (*p* < 0.05), suggesting older Asians had worse predicted prognosis (Fig. 2c). However, these comparisons are limited and likely invalid, confounded by much smaller sample sizes biased towards a greater proportion of high grade tumors among Asian patients. This serves as a cautionary tale of carefully examining whether it is reasonable to compare molecular subtypes or signatures by individual clinical characteristics. The available molecular datasets reflect patients diagnosed from 1977 to 2013 (median 1994) across a range of care settings, many often in specialist referral cancer centers, which may be more likely to treat more aggressive and later stage cancers. Some have many years of follow-up providing valuable opportunities for survival analysis; however, it is important to acknowledge that treatment approaches have changed substantially over the years meaning that therapy regimes, and thus outcomes, may be very different for more recently diagnosed patients.

Recent population-based cancer registry studies^7–9^ have shown an increase in the incidence rates of ER+ tumors, this pattern was also apparent in the publicly available datasets (Fig. 2d). However, the proportion of ER+ tumors is consistently lower in public gene expression datasets for each decade compared to data from Surveillance, Epidemiology, and End Results (SEER) Program (Fig. 2d). Similarly, the proportion of grade 1 tumors is lower in publicly available gene expression datasets than reported by SEER (Fig. 2e). Very limited numbers of tumors with published gene expression data and grade information are currently available from the 2010s for contemporary comparisons with the latest population data.

## Discussion

This study highlights the challenges of incomplete clinical and epidemiological information in many studies, the issue of high variation between datasets and likelihood of confounding due to limited annotation and bias in the patient population from whom the samples analyzed were taken. Some datasets more closely represent the wider or specific populations than others, but overall, they tend to be enriched for high grade, ER-negative tumors which may limit the applicability of any conclusions derived from these resources to the source populations that the breast cancers originate. The underlying sources of this bias observed in the public datasets are likely numerous and varied, primarily influenced by methodological requirements in earlier years for high tumor content and volumes for genetic profiling studies and sample availability rather than study design—causing a higher proportion of high grade ER-negative tumors than normally would be observed^10^. We recently highlighted that technological advances now enable reliable gene expression profiling of formalin fixed paraffin-embedded samples due to less stringent RNA extraction requirements^11^, but most of the datasets included in this study utilized fresh frozen research samples collected in addition to routine clinical samples.

The breast cancer research community is fortunate to have much larger in silico resources in the public domain for reanalysis compared to other malignancies. It is hoped that molecular datasets generated in the future will be more complete for demographic and clinical information. Most of these datasets are comprised of women of European descent, but it will also be necessary to ensure that under-represented groups such as women of African and Asian descent are appropriately represented if we are to consider molecular epidemiology of breast cancer at a global level to ensure precision medicine is accessible to all. Although it is nearly 20 years since the minimum information about a microarray experiment MIAME guidelines were introduced^12^ and reanalysis of publicly available data has moved on a long way since then, the guidelines on what information should be provided with datasets has not really changed. It does not seem unreasonable that along with mandating data availability, journals should begin insisting that minimum patient and tumor clinicopathologic information is provided (such as sex, age, and race or hospital, along with histologic grade, nodal status, ER, and HER2 status) for all samples for studies to be published. Whether and how researchers and patients could be incentivized to generate more representative datasets is perhaps more contentious, although it has been shown that minorities are often under-represented in clinical trials and steps can be taken to address this^13^. We recognize that there are social and historical factors that may affect participation of minority communities in such efforts, but it is incumbent upon the scientific community to make appropriate efforts to recruit these populations.

Tumor gene expression datasets for all cancer types will continue to proliferate in the public domain, providing a valuable resource to generate and test hypotheses for individual genes or signatures. However, researchers should be well aware of wide variations in terms of size, patient characteristics, and molecular composition of datasets and that they do not necessarily reflect the source population diagnosed cancers.

## Methods

### Dataset selection and processing

A total of 70 datasets, representing 16,130 breast carcinomas (Summarized in Supplementary Data Set 1) were identified in the public domain when restricting the search to those studies representing a minimum of 50 breast cancer patients with primary invasive breast tumors^14^. Datasets were generated on 15 different gene expression platforms, but Affymetrix GeneChips dominated (88%). The datasets were downloaded from ArrayExpress or NCBI Gene Expression Omnibus. Where available, raw.CEL files were processed using Custom CDF^15^ to Entrez gene IDs and fRMA normalized^16^. Preprocessed data for other platforms was utilized with multiple probes for the same gene averaged. Estimations of molecular subtype classifications and prognostic gene signatures were calculated using geneFu^17^. To consider the robustness of our meta-analysis approach, the 573 tumors from TCGA that had both RNAseq and Agilent microarray data were assessed for concordance (Supplementary Fig. 1).

### Reporting summary

Further information on research design is available in the Nature Research Reporting Summary linked to this article.

## Supplementary information

Supplementary Figure 1

Supplementary Dataset 1

Reporting Summary

## Data Availability

The data analysed during this study are described in the following data record: 10.6084/m9.figshare.c.4364174^14^. All of the gene expression datasets analyzed in the study are already publicly available, and their accession numbers and original publication references are listed in the Supplementary Data Set 1 included with the data record^14^.
