# Supplementary figures and images for: Breast cancer gene expression datasets do not reflect the disease at the population level
Source: NPJ Breast Cancer. 2020 Aug 25;6:39. doi: 10.1038/s41523-020-00180-x (PMC7447772; doi:10.1038/s41523-020-00180-x)

### PAM50

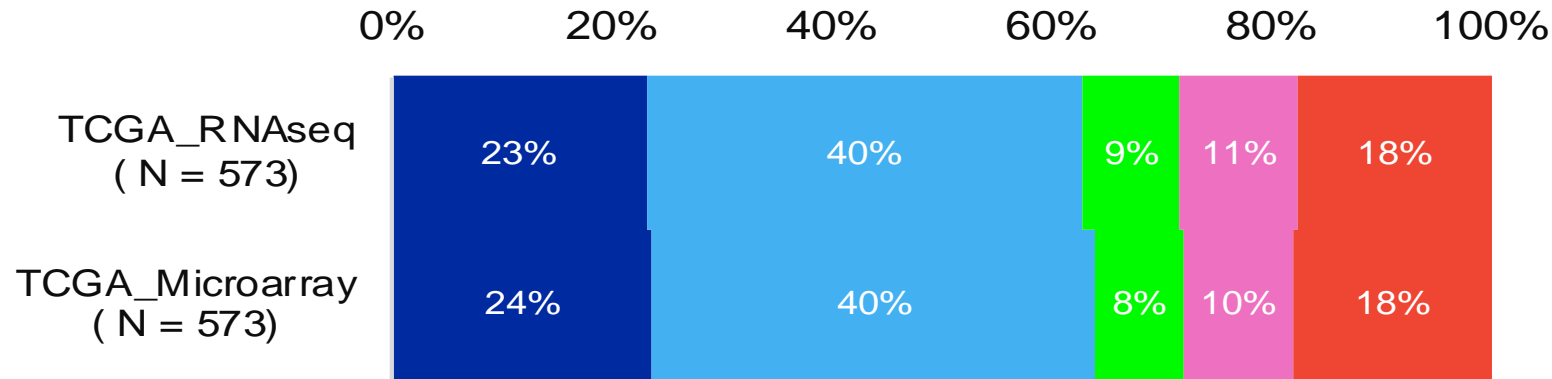

### SSP2006

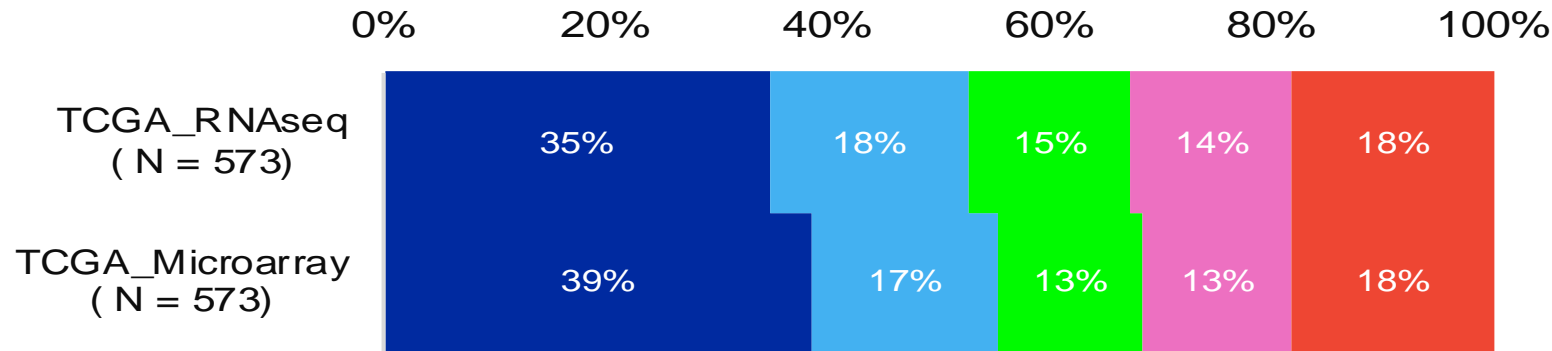

### SSP2003

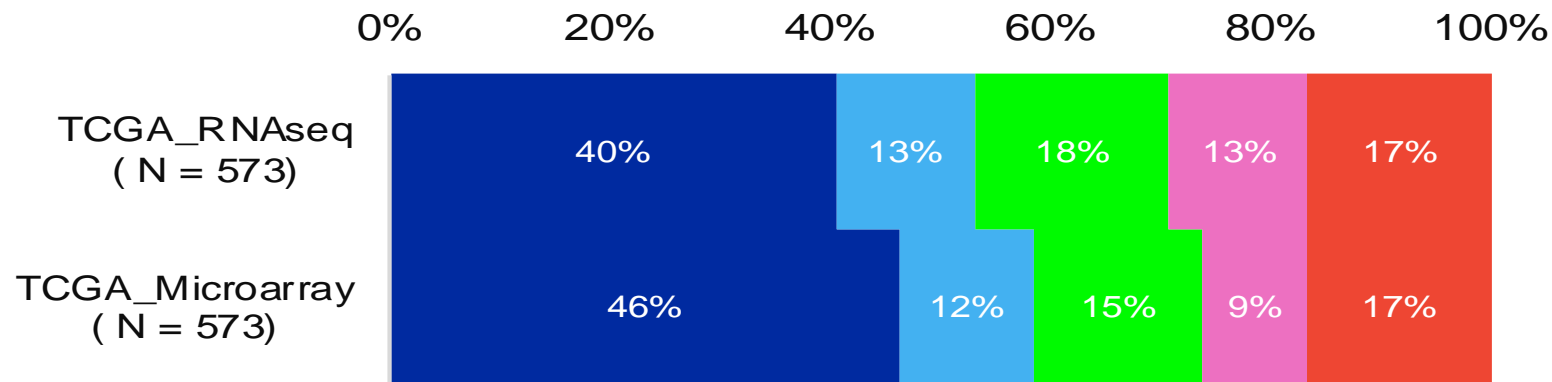

■ Luminal A ■ Luminal B ■ Normal-Like ■ HER2-Enriched ■ Basal-Like

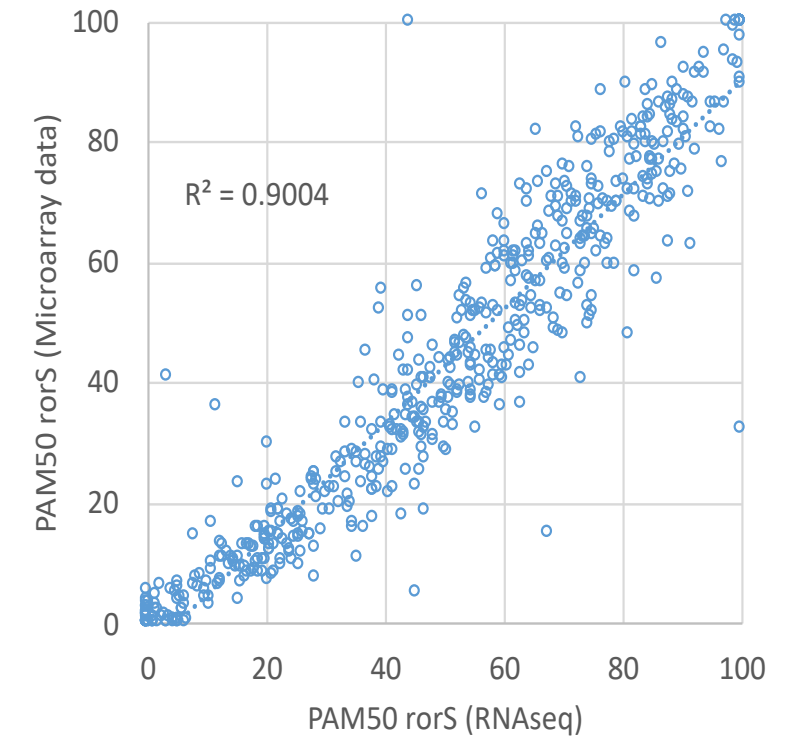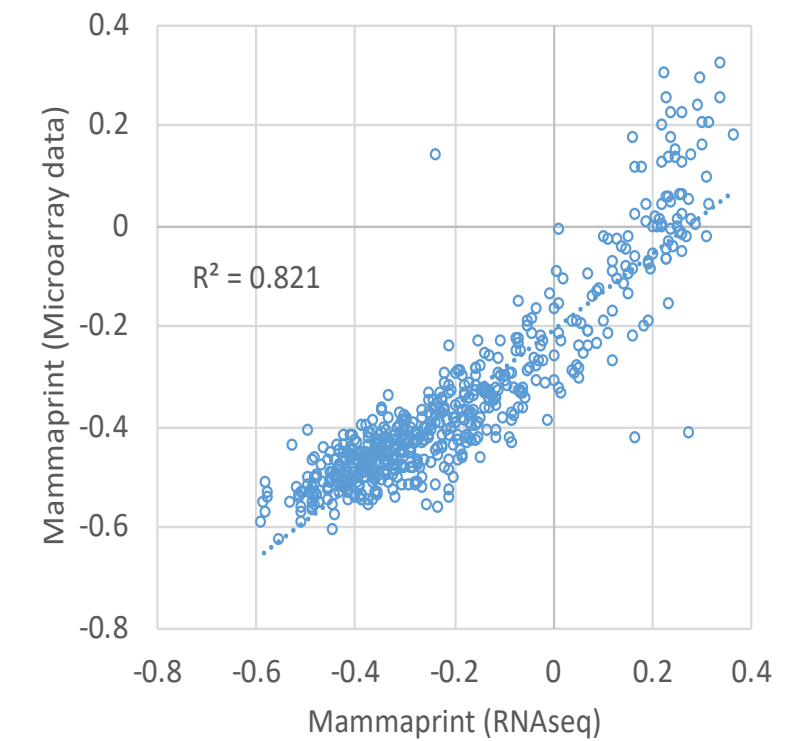

Supplement: Supplementary file 1 — Supplementary Figure 1 [file 41523_2020_180_MOESM1_ESM.pdf]
